# Murid Gammaherpesvirus Latency-Associated Protein M2 Promotes the Formation of Conjugates between Transformed B Lymphoma Cells and T Helper Cells

**DOI:** 10.1371/journal.pone.0142540

**Published:** 2015-11-06

**Authors:** Diana Fontinha, Filipa B. Lopes, Sofia Marques, J. Pedro Simas

**Affiliations:** Instituto de Medicina Molecular e Instituto de Microbiologia, Faculdade de Medicina, Universidade de Lisboa, Lisboa, Portugal; University Medical Center of the Johannes Gutenberg University of Mainz, GERMANY

## Abstract

Establishment of persistent infection in memory B cells by murid herpesvirus-4 (MuHV-4) depends on the proliferation of latently infected germinal center B cells, for which T cell help is essential. Whether the virus is capable of modulating B-T helper cell interaction for its own benefit is still unknown. Here, we investigate if the MuHV-4 latency associated M2 protein, which assembles multiprotein complexes with B cell signaling proteins, plays a role. We observed that M2 led to the upregulation of adhesion and co-stimulatory molecules in transduced B cell lines. In an MHC-II restricted OVA peptide-specific system, M2 polarized to the B-T helper contact zone. Furthermore, it promoted B cell polarization, as demonstrated by the increased proximity of the B cell microtubule organizing center to the interface. Consistent with these data, M2 promoted the formation of B-T helper cell conjugates. In an in vitro competition assay, this translated into a competitive advantage, as T cells preferentially conjugated with M2-expressing B cells. However, expression of M2 alone in B cells was not sufficient to lead to T cell activation, as it only occurred in the presence of specific peptide. Taken together, these findings support that M2 promotes the formation of B-T helper cell conjugates. In an in vivo context this may confer a competitive advantage to the infected B cell in acquisition of T cell help and initiation of a germinal center reaction, hence host colonization.

## Introduction

Gammaherpesviruses establish life-long persistent infections and are highly prevalent in the human population. Latent infection of circulating memory B cells is crucial to persistence and hence disease ontogeny. To access the memory B cell compartment gammaherpesviruses, such as Epstein-Barr virus (EBV) and murid herpesvirus-4 (MuHV-4), take advantage of germinal center (GC) reactions [1–7]. In the case of MuHV-4, at the latency peak (14dpi), it has been estimated that 70% of the infected B cells have a GC phenotype [8], which suggests some modulation of this route by the virus.

T cell help is critical for the initiation of a GC reaction in T cell-dependent immune responses. Before engaging in a cognate interaction with a B cell that will lead to its activation, proliferation and establishment of a GC [9], T helper (T_H_) cells scan for the highest affinity with particular antigen-presenting cells (APC). Such transient interactions occur in the border area between follicles and T cell zones and are mediated by adhesion molecules, resulting in the formation of B-T_H_ cell conjugates. Upon peptide recognition, the formation of an organized signaling structure, the immunological synapse (IS), takes place [10]. This process has been shown to be highly dynamic as T cells can interact with several APCs simultaneously, selectively polarizing towards the strongest stimulus [11].

MuHV-4 course of infection in the spleen has been recently characterized [12]. The virus first infects macrophages that provide access to marginal zone B cells. These, in turn, relocate to the white pulp where the virus is transferred to follicular dendritic cells (DCs). MuHV-4 then reaches follicular B cells, which are able to participate in a GC reaction. At this pre-GC stage, the ability of the infected follicular B cells to attract T cell help would be a major advantage for these viruses. In fact, importance of T cell help is reflected in studies that show defects in in vivo B cell activation [13] or demonstrate lower latency levels in the absence of CD4^+^ T cells [14, 15] or T follicular helper cells (T_FH_) [16].

To investigate if MuHV-4 had the ability to modulate B-T_H_ cell interactions, the M2 protein was chosen as a potential candidate. It is one of the few viral proteins that is expressed during the latency phase [17]. It is a putative functional homologue of the transmembrane proteins LMP1 and LMP2A encoded by EBV, and K1 and K15 encoded by Kaposi sarcoma-associated herpesvirus (KSHV), which either mimic or interfere with BCR signaling [18–20]. Contrarily to these proteins, M2 is a soluble cytoplasmic protein. Its expression has been demonstrated in B cells [17] where it localizes to juxtamembranar areas of the cell, a process that relies on a C-terminal proline-rich SH3 binding region of M2 and its interaction with Src family kinases [21–23]. It contains two phosphosites (tyrosine residues Tyr^120^ and Tyr^129^), that are constitutively phosphorylated by Src family kinases [18, 19], that form an unconventional immunoreceptor tyrosine activation motif (ITAM). This ITAM is implicated in M2 ability to work as a modulator protein, coordinating the assembly of multiprotein complexes with cell signaling proteins, namely NCK1, Vav1, PLCγ2, the tyrosine phosphatase SHP2 and the p85α subunit of PI3K [21]. Therefore, just like its putative functional homologues, M2 mediates the assembly of specific signalosomes. On the one hand, M2 interaction with the Fyn/Vav pathway leads to the activation of Vav1 [18, 19]. Furthermore, M2 drives tyrosine phosphorylation of PLCγ2. On the other hand, M2 expression leads to inhibition of AKT activation upon BCR stimulation. M2 is also known to drive IL-10 dependent B cell proliferation and differentiation [24]. IL-10 production increase upon expression of M2 in B cells is a consequence of the induction of IRF4 via the NFAT pathway [25]. In vivo, M2 is important for latency establishment and efficient proliferation of infected GC B cells [22, 26, 27]. Infection of mice with a virus encoding the M2Y mutant (designated vM2Y, with Tyr^120^ and Tyr^129^ residues mutated to phenylalanine) results in a delay in latency establishment [18]. More recently, it has been shown that in vivo only Tyr^129^ is critical for reactivation from latency and plasma cell differentiation [28].

Here, we investigated if the latency associated protein M2 encoded by MuHV-4 is able to modulate B-T_H_ cell interaction. We observed that B cells expressing M2 are more prone to recruit T cell help, promoting the formation of B-T_H_ cell conjugates. Furthermore, we showed that this function was dependent on the ITAM of M2, hence dependent on the ability to assemble multiprotein complexes with B cell signaling proteins. Overall, in vivo, this may confer a competitive advantage to the infected B cell in the recruitment of T cell help and initiation of a GC reaction, therefore host colonization.

## Results

### Expression of the Latently Associated Protein M2 Leads to the Upregulation of Adhesion and Co-Stimulatory Molecules in B Cells

**Fig 1:**
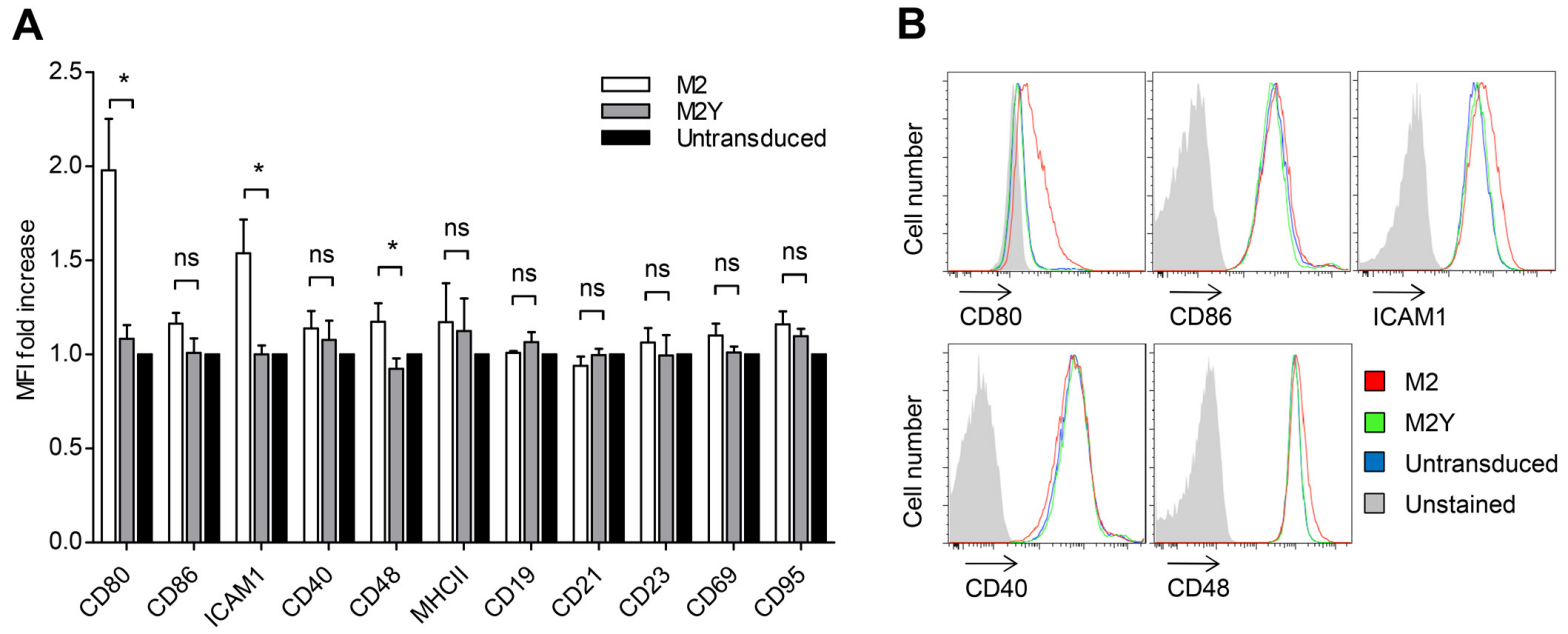
M2 expression leads to the upregulation of adhesion and co-stimulatory molecules in B cells. (A) Fold increase of the mean fluorescence intensities (MFI) of several surface molecules, relative to untransduced A20 B cells. A20 B cells (black bars) and A20 B cells stably expressing M2 (open bars) or M2Y (grey bars) were stained with fluorescently labelled antibodies and the surface expression of the indicated molecules was analyzed on a LSR Fortessa flow cytometer. Bars represent the mean of three independent experiments. Error bars represent standard error of the mean. Statistical significance was evaluated using a one-tailed Students t-test. (B) Representative FACS histogram plots for the indicated surface molecules.

In order to assess if M2 plays a role in B-T_H_ cell interactions we started by analyzing the influence of the viral protein on the levels of selected B cell surface molecules, some of which have been shown to be involved in IS formation [10, 29, 30]. In parallel, we used an M2 mutant protein (M2Y), which has the tyrosine residues 120 and 129 mutated to phenylalanines. These residues are not phosphorylated and the ability to assemble multiprotein complexes with B cell signaling proteins, and its downstream effects, are impaired [18, 19, 21]. A20 B cells stably expressing M2 or the M2Y mutant were stained for the indicated markers and analyzed by flow cytometry (Fig 1). Untransduced A20 B cells were used as a negative control. Fig 1A represents the mean fluorescence intensity (MFI) fold change of M2 or M2Y relative to that of the control. Levels of CD80, a co-stimulatory molecule [10, 29, 30], was two-fold higher in M2-expressing B cells than in the control. The cell adhesion molecules ICAM1 and CD48 [10, 29, 30] were 1.5- and 1.2-fold upregulated, respectively. These differences can be observed as a shift in fluorescence intensity in the representative FACS histograms (Fig 1B). This upregulation was specific as M2 did not significantly alter the levels of CD86, CD40, MHCII, CD19, CD21, CD23, CD69, and CD95. M2Y-expressing B cells did not show increased levels of any of these molecules, corroborating the use of M2Y as a negative control. Upregulation of CD80 and ICAM-1, as well as of CD86, was also observed in M2-expressing independent B cell lines (S1 Fig).

These results show that expression of M2 upregulates B cell co-stimulatory and adhesion molecules that participate in the formation of an IS. This function is dependent on tyrosine phosphorylation at the ITAM, hence related to the ability of M2 to assemble multiprotein complexes with B cell signaling proteins.

### M2 Polarizes to the B-T_H_ Contact Zone and Increases B Cell MTOC Polarization

During T cell scanning that takes place in the border area between follicles and T cell zones, B and T_H_ cells interact with each other forming conjugates. Upon cognate interaction and IS formation, cells undergo morphological and cytoskeletal changes, which result in the polarization of the microtubule organizing center (MTOC) [31–33], cellular organelles and signaling machinery to the contact zone [30, 34]. This polarization is reciprocal, as B cells also have the ability to polarize their MTOC and endocytic/exocytic compartments to the interface [35].

**Fig 2:**
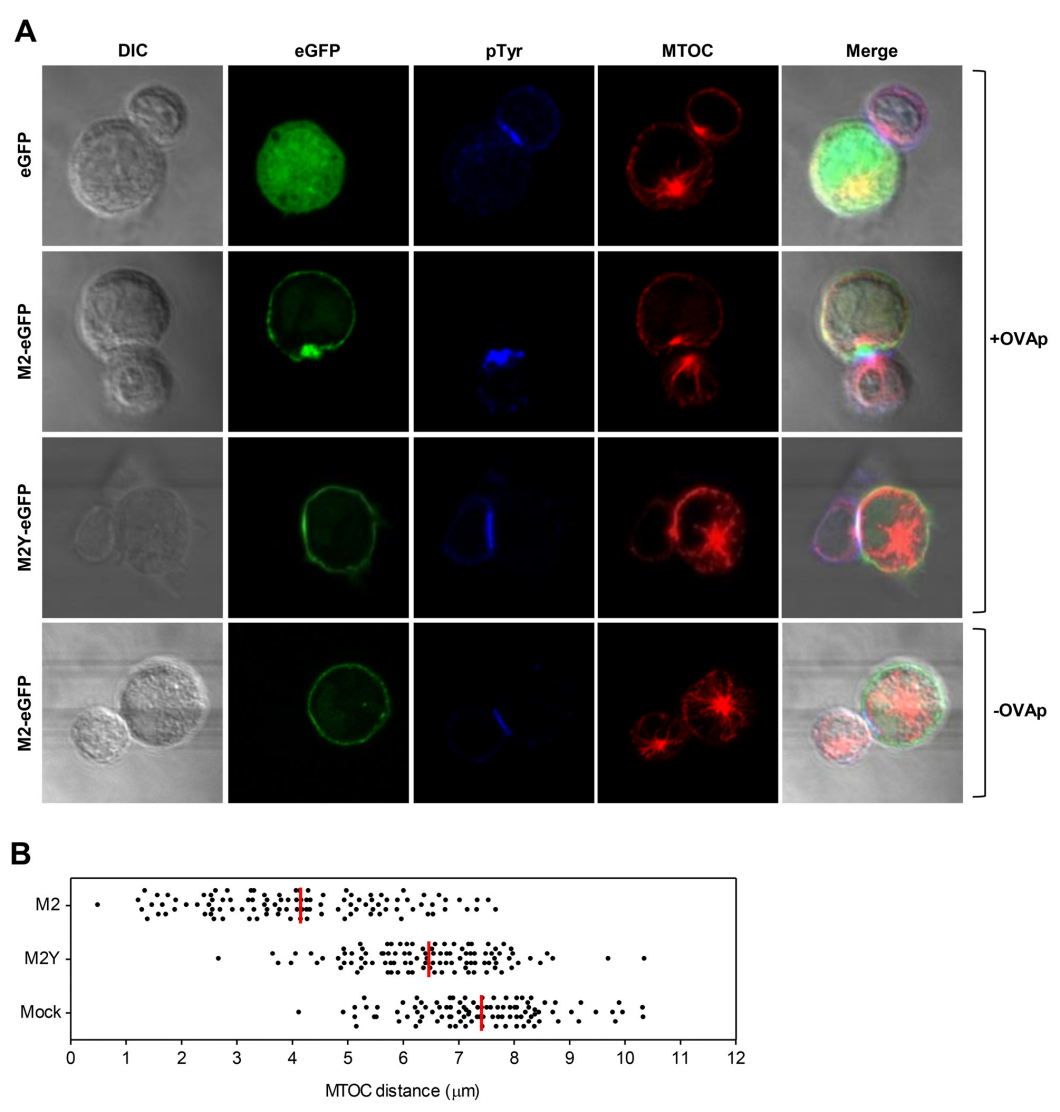
M2 polarizes to the B-T_H_ contact zone and increases B cell MTOC polarization. A20 B cells transiently expressing M2-eGFP, M2Y-eGFP or eGFP (green) were pulsed (three upper panels), or not (bottom panel), overnight with 10μM of OVA peptide (OVAp) and incubated with OVAp-specific CD4^+^ T cells for 15 minutes. Cells were fixed and stained for pTyr (blue), and for MTOC with an anti-α-tubulin antibody (red). (A) Representative images of M2 and B cell MTOC polarization to the contact zone. (B) Quantification of MTOC distance. Distance of the B cell MTOC to the contact zone was measured from a total of 100 conjugates from three independent experiments. The mean distance is represented as a red line. Statistical significance between groups was analyzed by one way ANOVA, with p<0.0001.

To address the effect of M2 expression in the formation of B-T_H_ cell conjugates, we used a MHC-II restricted cell system in which mouse A20 B cells, pulsed with ovalbumin peptide (OVAp), were conjugated with primary mouse OVAp-specific TCR-transgenic CD4^+^ T cells purified from Balb/c DO11.10 mice [36]. We first assessed M2 cellular distribution in the context of B-T_H_ cell interaction by expressing M2 and M2Y eGFP-tagged proteins. Furthermore, to address B cell polarization, we measured the B cell MTOC distance to the contact zone (Fig 2). To that end, mouse A20 B cells transiently expressing M2-eGFP, M2Y-eGFP or eGFP (mock) were pulsed, or not, with 10μM of OVAp and incubated with T_H_ cells for 15min. Cells were then fixed and stained for pTyr (blue), and α-tubulin (red) (Fig 2A). Using this system we observed that M2 polarizes to the contact zone as visualized by the accumulation of GFP signal at the interface. This polarization is dependent on the integrity of the ITAM as M2Y remained localized in juxtamembrane areas of the cell (Fig 2A). Next, the distance of the B cell MTOC to the contact zone was quantified (Fig 2B). M2 expression decreased B cell MTOC distance to the contact zone in 3.3μm compared to the mock control, i.e. it increased its polarization toward the conjugating T_H_ cell. Nonetheless, this polarization was incomplete, as it has been previously described for B cells [35]. M2Y also caused a statistically significant difference compared to the mock control. However, this was approximately three times less than the difference promoted by the wild type protein.

We conclude that M2 polarizes to the B-T_H_ cellular interface in a MHC class II-restricted OVAp-specific manner, a process that is dependent on the protein phosphosites. Results also show that M2 promotes B cell polarization, as its expression increased MTOC proximity to the contact zone.

### M2 Promotes the Formation of B-T_H_ Cell Conjugates

**Fig 3:**
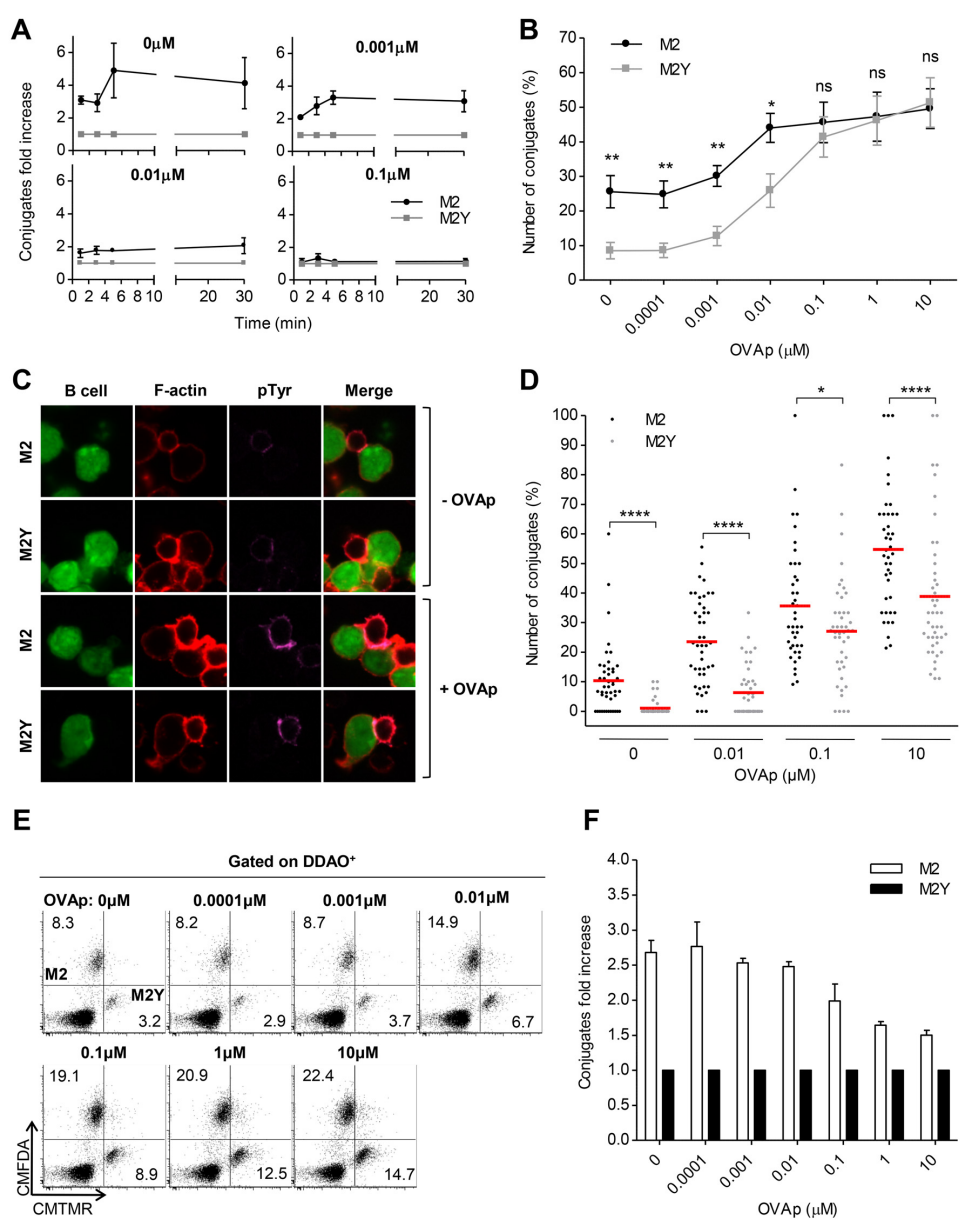
M2 promotes the formation of conjugates between B and T_H_ cells. A20 B cells stably expressing M2 or M2Y were pulsed overnight, or not, with different concentrations of OVA peptide (OVAp) and incubated with OVAp-specific CD4^+^ T cells at a 2:1 ratio, for the indicated time periods. Prior to incubation B and T cell populations were incubated with the live dyes CMFDA, CMTMR, and DDAO, respectively, to allow their discrimination. (A) Fold increase of the number of conjugates formed with M2- (black circles) or M2Y- (grey squares) expressing B cells relative to M2Y condition, determined by flow cytometry. Conjugate formation was evaluated as the percentage of CMFDA^+^DDAO^+^ events in the total DDAO^+^ population. (B) Percentage of conjugates after 30min of incubation upon variation of the OVAp concentration. (A) and (B) Symbols represent the mean of 4 independent experiments. (C) Representative images of pTyr polarization to the contact zone. After 30min of incubation, cells were fixed and stained for F-actin with TRITC-phalloidin (red), and for pTyr (purple). Images are from one representative experiment out of three. (D) Percentage of conjugates per field after 30min of incubation, as determined by confocal microscopy, upon variation of the OVAp concentration. Conjugate count was blind and based on B-T_H_ cell contact and pTyr polarization to the contact zone. A total of 45 images were taken per sample from three independent experiments. Only images with a minimum of three T cells were considered for analysis. (E) Representative FACS plots of in vitro competition assay for each OVAp concentration. M2-expressing B cells, M2Y-expressing B cells and CD4^+^ T cells were mixed at a 1:1:1 ratio and incubated for 30min. Conjugates were analyzed as the percentage of CMFDA^+^DDAO^+^ (M2 conjugates) or CMTMR^+^DDAO^+^ (M2Y conjugates) events in the total DDAO^+^ population. Percentage of T cells conjugating with M2 or M2Y-expressing B cells is indicated in the respective quadrant. (F) Fold increase of the number of conjugates formed with M2- (open bars) or M2Y- (filled bars) expressing B cells relative to M2Y condition is represented in the graph. In flow cytometry experiments error bars represent standard error of the mean. Statistical significance between groups was evaluated by a one-tailed unpaired Student’s t test. In confocal microscopy experiments statistical significance of the difference between groups was evaluated by a Mann-Whitney U test.

B and T_H_ cells interact with each other forming conjugates, a process that is mediated by adhesion molecules. Given that M2-expressing B cells showed increased levels of such molecules, we investigated if M2 had an impact in B-T_H_ cell conjugate formation (Fig 3). We used the MHC class II-restricted OVAp-specific system described above. Mouse A20 B cell lines, stably expressing M2 or M2Y, were loaded with CMFDA live dye and pulsed with increasing concentrations of OVAp. These were then incubated with T_H_ cells (loaded with DDAO live dye), in a 2:1 ratio, for 1, 3, 5 or 30 minutes. Conjugate formation was next assessed by flow cytometry, based on the percentage of CMFDA^+^DDAO^+^ events in the total DDAO^+^ population. Fig 3A shows the fold increase of the percentage of T cells conjugating with M2-expressing B cells (black circles), relative to the percentage of T cells conjugating with M2Y-expressing B cells (grey squares), against increasing incubation times. In the absence of peptide or with low concentrations of peptide, such as 0.001μM, conjugate formation with M2-expressing B cells increased with incubation time reaching a three-fold plateau that was maintained after 30 minutes of incubation. Fig 3B shows the percentage of M2 (black circles) or M2Y (grey squares) conjugates, after 30 minutes of incubation, upon variation of the OVAp concentration. Conjugate formation occurred in a peptide dose-dependent manner. In the absence of peptide, less than 10% of the T_H_ cells conjugated with M2Y-expressing B cells. In contrast, the integrity of the phosphosites resulted in a three-fold increase in conjugate formation with M2-expressing B cells. Up to 0.01μM M2 was able to promote conjugate formation. However, in the presence of high concentrations of OVAp, M2 did not influence conjugate formation. We conclude that M2 promotes conjugate formation in the absence or with low concentrations of OVAp. Such ability is dependent on the phosphosites responsible for binding to B cell signaling proteins.

To further address conjugate formation, we analyzed B-T_H_ cell interaction by confocal microscopy, including a marker for phosphorylated tyrosines (pTyr). This is indicative of signaling and formation of a functional immunological synapse but, in this particular case, can be also associated with constitutive phosphorylation of the tyrosine residues of M2, and the consequent phosphorylation of Vav1 and PLCγ2 [18, 19, 21]. Using the MHC class II-restricted OVAp-specific system, M2- or M2Y-expressing B cell lines, loaded with different concentrations of peptide, and T_H_ cells were incubated in a 2:1 ratio, for 30 minutes. Prior to incubation, B cells were loaded with CMFDA live dye to allow their visualization. Cells were also stained for F-actin, to allow T cell, as well as B-T_H_ cell interaction, visualization. Conjugate count was blind and based on B-T_H_ cell contact and pTyr (purple) polarization to the contact zone (Fig 3C). Fig 3D shows the percentage of T cells conjugating with either M2- (black circles) or M2Y-expressing (grey circles) B cells. Corroborating the previous results, confocal microscopy revealed that M2 expression in B cells promoted conjugate formation with T_H_ cells, with pTyr polarization to the contact zone.

To understand whether the increase in conjugate formation translated into a competitive advantage, we performed an in vitro competition assay. T_H_ cells were simultaneously incubated with equal numbers of M2- and M2Y-expressing B cells in a 1:1:1 ratio. Both B cell populations were pulsed with the same OVAp concentration. Prior to incubation, the three populations were loaded with different live dyes to allow their discrimination (Fig 3E), as was performed above. Fig 3F shows the fold increase of the percentage of T cells conjugating with M2-expressing B cells (open bars) relative to the percentage of T cells conjugating with M2Y-expressing B cells (filled bars). On average, there were 2.5-fold more M2 conjugates when peptide concentration was low (up to 0.1 μM) or zero. This shows that T_H_ cells preferentially conjugated with B cells expressing the wild-type viral protein. To exclude the possibility of a cell line effect, all three experiments were repeated with independently generated B cell lines with similar results (S2 Fig).

Taken together, these data show that expression of M2 gives a competitive advantage to B cells by favoring the formation of conjugates with T_H_ cells, independently of specific antigen presentation.

### M2-Expressing B Cells Do Not Promote T Cell Activation in the Absence of Specific Peptide

**Fig 4:**
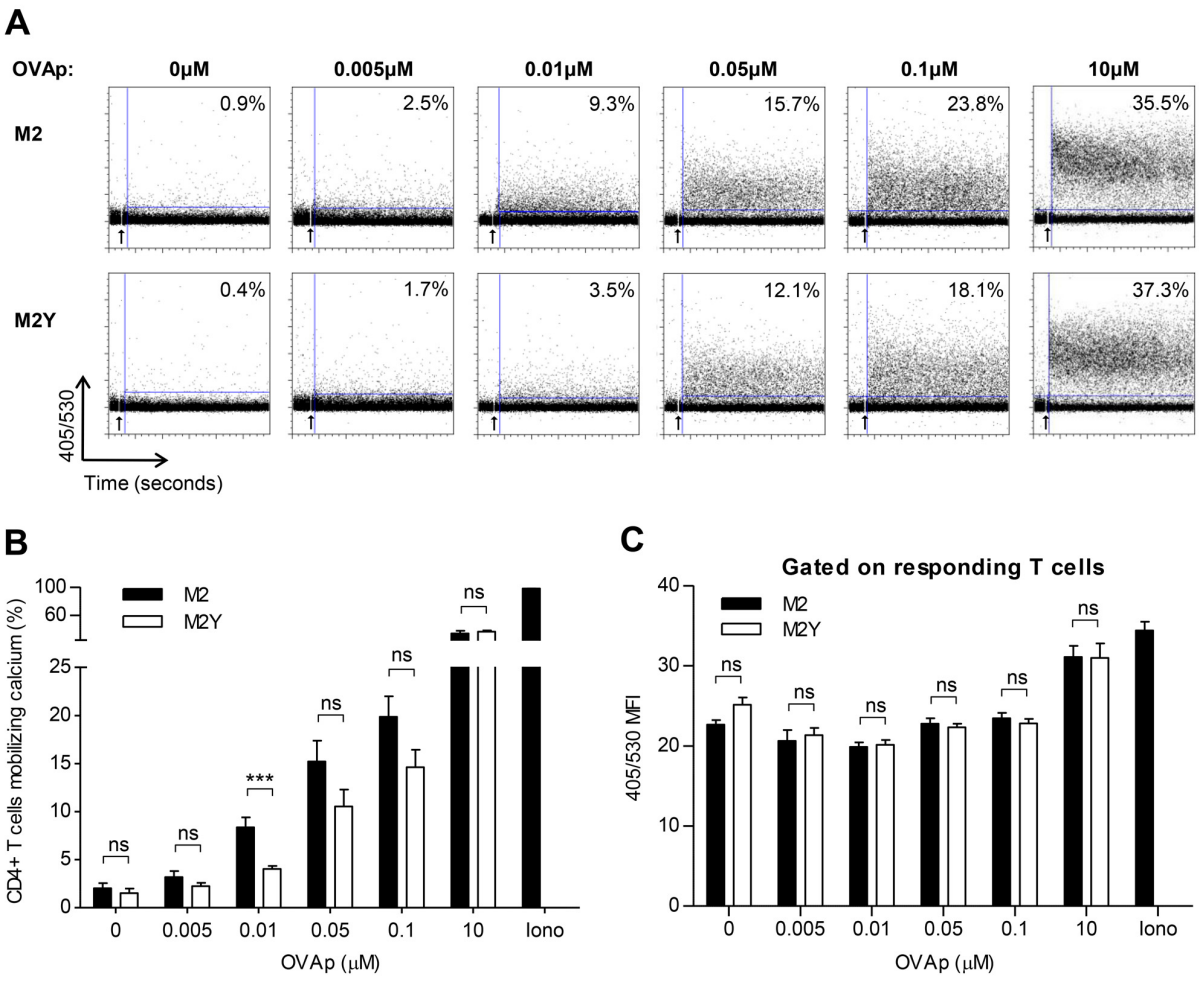
Calcium mobilization in T_H_ cells conjugated with M2-expressing B cells requires specific peptide presentation. A20 B cells stably expressing M2 or M2Y were pulsed overnight, or not, with different concentrations of OVAp and incubated with OVAp-specific CD4^+^ T cells for 5 min. Prior to conjugation T cells were loaded with the calcium indicator Indo-I. Ionomycin was used as a positive control. (A) Representative FACS plots of calcium measurements. A baseline was established by acquiring unstimulated T_H_ cells alone, for approximately two minutes. Calcium fluxes were then measured on a MoFlow cytometer for 18 minutes (indicated by an arrow) and were based on the 405/530 emission ratio over time. 405/530 mean fluorescence intensity (MFI) is shown. (B) Average of the percentage of CD4^+^ T cells mobilizing calcium when conjugated with M2-expressing (filled bars) or M2Y-expressing (open bars) B cells. (C) 405/530 MFI average within responding T cells. Graphs show results from three to seven experiments. Error bars represent standard error of the mean. Statistical significance of the difference between groups was evaluated by a one-tailed unpaired Student’s T test.

Cognate B-T_H_ cell interaction results in IS formation. This is a bidirectional communication system whereby signals transmitted at the contact interface can lead to activation of both target and effector cells. Thus, we next investigated if the competitive advantage in interaction with T helper cells, conferred by M2 expression in B cells, resulted in T cell activation. Using the MHC class II-restricted OVAp-specific system we assessed intracellular calcium concentration increase by flow cytometry (Fig 4), as a marker of T cell activation. Prior to incubation, T_H_ cells were loaded with the calcium indicator Indo-1, which changes its emission spectrum by chelating free calcium ions. As a result, the intensity of the emission at 405nm increases and, simultaneously, the intensity of the emission at 530nm decreases. Therefore, the 405/530 ratio provides a measure of intracellular calcium increase (Fig 4A). For each sample, a baseline was established by acquiring unstimulated T_H_ cells alone, for approximately two minutes [37]. Then, the effect of incubation with M2- or M2Y-expressing B cells on T_H_ cell intracellular calcium increase was assessed (indicated by an arrow in Fig 4A). Values shown on Fig 4A refer to the percentage of responding T_H_ cells, i.e. T_H_ cells mobilizing calcium from one representative experiment. Ionomycin was used as a positive control, leading to activation of more than 97% of the T cell population. The percentage of responding T_H_ cells increased with the OVAp concentration (Fig 4B). In the absence of peptide M2 did not promote intracellular calcium increase. When pulsed with 0.01μM of OVAp, M2-expressing B cells led to an increase in the number of T_H_ cells mobilizing calcium, compared to M2Y-expressing B cells. To assess if M2-expressing B cells were also able to promote stronger individual responses, we quantified the 405/530 ratio MFI of responding T_H_ cells (Fig 4C). M2 and M2Y samples showed no statistically significant differences in MFI demonstrating that, even though there were more T_H_ cells mobilizing calcium, there was no difference in the magnitude of individual responses. This experiment was repeated with independent B cell lines, for some OVAp concentrations, with similar results (S3A Fig).

**Fig 5:**
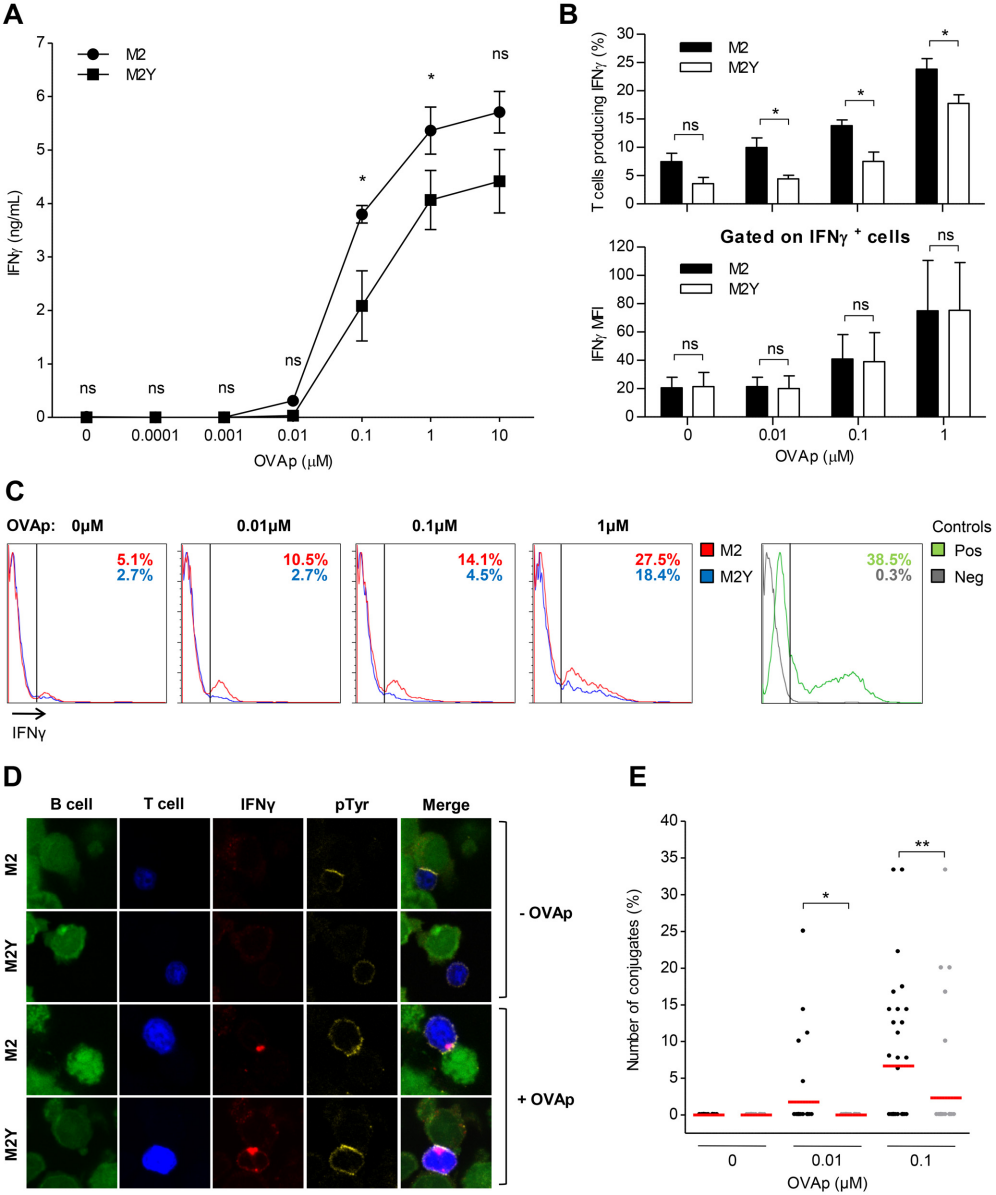
IFN-γ production in T_H_ cells conjugated with M2-expressing B cells requires specific peptide presentation. A20 B cells stably expressing M2 or M2Y were pulsed overnight, or not, with different concentrations of OVA peptide (OVAp) and incubated with OVAp-specific CD4^+^ T cells. (A) Extracellular IFN-γ after 20h of incubation. After incubation the supernatant was recovered and analyzed by sandwich ELISA to determine IFN-γ concentration. Supernatants of three independent experiments were tested in duplicate. (B) Upper panel: percentage of T cells producing IFN-γ after 5h of conjugation in the presence of brefeldin A (BFA). Cells were fixed and stained for CD4 with anti-CD4-APC, and for IFN-γ with anti-IFN-γ-PE, and analyzed on a FACS Calibur. Lower panel: IFN-γ mean fluorescence intensity (MFI) of responding T cells. Average of three independent experiments is shown. Statistical significance of the difference between groups was evaluated by a one-tailed unpaired Student’s t test. (C) Representative FACS plots of intracellular IFN-γ production. (D) Representative confocal images of IFN-γ polarization to the contact zone. Prior to incubation B and T cells were labelled with CMFDA (green) and CMAC (blue) live dyes, respectively. Cells were incubated for 2.5h, fixed and stained for IFN-γ (red), and for pTyr (yellow). Images are from one representative experiment out of three and were obtained using a Zeiss LSM 510 META microscope. (E) Quantification of conjugates with IFN-γ polarization per image. Conjugates were evaluated by confocal microscopy based on B-T_H_ cell contact, and IFN-γ polarization (red). 45 to 55 images were acquired per sample from three independent experiments. Only images with a minimum of three T cells were considered for analysis. Statistical significance of the difference between groups was evaluated by a Mann-Whitney U test.

To further investigate if M2 expression in B cells could have an effect in promoting T_H_ cell activation we quantified T cell IFN-γ production and release by ELISA (Fig 5A). After 20h of incubation IFN-γ concentration was measured on culture supernatants by sandwich ELISA. In the absence of peptide we could not detect IFN-γ. For 0.1μM and 1μM of OVAp, M2 expression led to an increase in IFN-γ concentration, compared to the M2Y condition. The IFN-γ production increase observed by ELISA could be due to an increase in production per cell or due to an increase in the number of activated T_H_ cells. To discriminate between these two possibilities, we analyzed IFN-γ production by flow cytometry (Fig 5B and 5C). B and T_H_ cells were incubated in the presence of brefeldin A (BFA) to inhibit protein secretion. As shown in the upper panel of Fig 5B, M2-expressing B cells did not significantly promote IFN-γ production in the absence of peptide. From 0.01μM to 1μM of OVAp concentrations, M2 expression led to an increase in the percentage of T_H_ cells producing IFN-γ, compared to M2Y. Measurement of IFN-γ MFI of responding T_H_ cells showed no significant differences between M2 and M2Y conditions (Fig 5B, lower panel). This can be confirmed in the representative FACS plots (Fig 5C). Therefore, as observed in the calcium measurement experiment, we conclude that the increased IFN-γ production resulted from an increase in the number of activated T_H_ cells, and not from stronger individual responses.

We also assessed IFN-γ polarization to the contact zone by confocal microscopy (Fig 5D). Quantification of the percentage of T_H_ cells, conjugating with either M2- (black circles) or M2Y-expressing (grey circles) B cells, that polarize IFN-γ to the contact zone (Fig 5E), shows that there is a requirement for presentation of specific peptide. In the presence of peptide, conjugate formation with M2-expressing B cells resulted in more T_H_ cells activated compared to M2Y. This experiment was repeated with independent B cell lines, with similar results (S3B Fig).

Collectively, these experiments show that in the context of conjugate formation, expression of M2 in B cells does not promote T cell activation in the absence of specific peptide.

## Discussion

With this work, we further characterized the role of the latency associated M2 protein, involved in the assembly of multiprotein complexes with cell signaling proteins [21], and linked it to the modulation of B-T_H_ cell interactions. Our data supports a model where expression of MuHV-4 M2 protein competitively promotes interaction with T_H_ cells in vitro, in the context of a MHC class II-restricted OVAp-specific cellular system. This process was independent of presentation of specific antigen and may be the outcome of the upregulation of adhesion molecules observed in M2-expressing B cells. However, in the absence of specific peptide, conjugate formation with M2-expressing B cells did not result in increased intracellular calcium levels or IFN-γ production in T_H_ cells. Therefore, T cell activation upon conjugate formation was not driven by M2, but a product of cognate interaction. Nevertheless, concomitant with the increased number of conjugates, the number of activated T_H_ cells was higher in the presence of M2-expressing B cells.

B cell polarization has been rarely addressed in B-T_H_ cell interaction studies, although it has been described [35]. Here, we provided evidence of B cell polarization in B-T_H_ cell interaction. Collectively, B cell polarization and the upregulation of co-stimulatory molecules observed under M2 expression suggest a role for the viral protein in IS modulation.

Modulation of T-APC interaction, and more specifically IS, by a viral protein has been previously described. Protein Nef from Human Immunodeficiency Virus type 1 (HIV-1) has been shown to impair IS formation [38–41], as a means of optimizing the environment for HIV infection. In the *Gammaherpesvirinae* there are also two examples of impairment of B-T_H_ cell interactions and IS formation, namely K5 from Kaposi Sarcoma-associated Herpesvirus (KSHV) [42], and tyrosine kinase-interacting protein (Tip) from the T lymphotropic Herpesvirus Saimiri (HVS) [43]. In contrast, our results support a model where a viral protein promotes B-T_H_ cell interaction, instead of impairing it. This is concomitant with the high prevalence of infection in GC B cells. Such positive effect does not exclude the possibility that, at some point during infection, MuHV-4 impairs IS formation as a mechanism of immune evasion.

The inability of M2Y to promote B-T_H_ cell conjugate formation in vitro is in agreement with the delay in latency establishment observed in vivo with the recombinant M2Y virus [18]. However, this virus is still able to enter GC reactions, meaning that there are other players in this process. For example, the putative existence of a viral superantigen [44] may drive IS formation. Another possibility lies with the choice of B cells for latency establishment. This has been demonstrated not to be a stochastic event, in what concerns BCR specificity, and to be linked to the formation of GCs [45].

Despite the controversy regarding antigen dependence in the production of EBV-infected memory B cells, there is unequivocal evidence of the participation of EBV-infected B cells in the GC reaction [1, 2], as it is described for MuHV-4 [5–7]. However, the role that the virus plays in such process still lacks characterization. Although it is not clear yet, it is possible that KSHV also takes advantage of the GC reaction. Given that M2 is a putative functional homologue of LMP1 and LMP2A encoded by EBV, and K1 and K15 encoded by KSHV, our work sets the base for future studies with the mentioned proteins, in what regards T-APC interaction.

In this study we propose that the latency-associated M2 protein competitively promotes B-T_H_ cell interaction, a process that is dependent on the modulation of B cell signaling. In vivo, this ability to attract T cell help in a competitive manner during the T cell scanning process, carried out in secondary lymphoid organs, may account for the selection of infected B cells for GC initiation, a crucial step in latency establishment, hence host colonization.

## Materials and Methods

### Ethics Statement

The study accorded with the Portuguese official Veterinary Directorate (Portaria 1005/92), European Directive 2010/63/EU, and Federation of European Laboratory Animal Science Associations guidelines on laboratory animal welfare. It was approved by the Portuguese official veterinary department for welfare licensing (protocol AEC_2010_017_PS_Rdt_General) and by the IMM Animal Ethics Committee.

### Mice

8-week to 4-month old Balb/c TCR transgenic DO11.10 mice (kindly provided by Prof. Luís Graça, Instituto de Medicina Molecular) were used for purification of CD4^+^ T cells. Mice were bred and housed under specific pathogen-free conditions at Instituto de Medicina Molecular, Lisbon, Portugal. Mice were sacrificed by CO_2_ inhalation or cervical dislocation.

### Plasmids

pEGPF-M2, pEGFP-M2Y plasmids have been described [21]. To produce viral transduction particles for the creation of zeocin-resistant stable cell lines, M2 was cut from pMSCV-M2-IRES-GFP [19] with EcoRI and XhoI and subcloned to pMSV-K3-IRES-Zeo [20], replacing K3 and giving rise to pMSCV-M2-IRES-zeo. Similarly, pMSCV-M2Y-IRES-Zeo was constructed by excising M2Y from pMSCV-M2Y-IRES-GFP [19] and subcloning it as described for pMSCV-M2-IRES-Zeo. To produce viral transduction particles for the creation of zeocin-resistant, eGFP-expressing, stable cell lines, pMSCV-eGFP-M2-IRES-zeo and pMSCV-eGFP-M2Y-IRES-zeo plasmids were constructed, were eGFP is N-terminally fused to M2/M2Y. For that purpose, eGFP was amplified by PCR from pEGFP-N1 vector (Clontech). pEQPAM3 was the packaging vector used.

### Antibodies

Primary antibodies used for confocal microscopy were: anti-F-actin (TRITC-phalloidin—Sigma), anti-alpha tubulin (clone DM1A, Sigma-aldrich), anti-pTyr-AF647 (clone PY99, Santa Cruz Biotechnology), anti-IFN-gamma (clone AN-18, BD Biosciences). Secondary antibodies used for confocal microscopy were: anti-rat AF568 (Molecular Probes) and anti-mouse AF594 (Molecular Probes). Antibodies used for flow cytometry were: anti-TCR DO11.10 PE (clone KJ1.26, MBL-medical and biological lab), anti-CD4 AF405 (clone RM4-5, Invitrogen), anti-CD80 PB (clone 16-10A1, BioLegend), anti-CD86 PE-Cy7 (clone PO3, BioLegend), anti-CD54 (ICAM1) APC (clone YN1/1.7.4, BioLegend), anti-CD40 PE-Cy7 (clone 3/23, BioLegend), anti-CD48 APC (clone HM48-1, BioLegend), anti-MHC-II PB (clone M5/114.15.2, BioLegend), anti-CD19 APC-H7 (clone 1D3, BD Biosciences), anti-CD21 APC (clone 7G6, BD Biosciences), anti-CD23 PE (clone B3B4, BD Biosciences), anti-CD69 FITC (clone H1.2F3, BioLegend), anti-CD95 PE (clone Jo2, BD Biosciences), anti-IFN-γ PE (clone XMG1.2, BD Biosciences). Primary antibodies used for western blot were: anti-EGFP (Clontech) and anti-actin (polyclonal, Sigma-Aldrich). Secondary antibodies used for western blot were anti-rabbit (GE Healthcare) and anti-mouse (Jackson Immunoresearch) antibodies, conjugated with horse radish peroxidase (HRP).

### Cell Culture, Transfection and Transduction

HEK-293T cells were cultured in Dulbecco’s modified Eagle’s medium (DMEM) supplemented with 10% fetal bovine serum, 2mM glutamine, 100U/mL penicillin and streptomycin. A20 B cells were grown in RPMI 1640 supplemented as described above. In the case of zeocin-resistant stable cell lines, 4μg/mL of zeocin was added to the culture. Conjugates were incubated in RPMI 1640 supplemented with 5% fetal bovine serum, 2mM glutamine, 100u/mL penicillin and streptomycin and 10mM HEPES. In the case of the analysis by flow cytometry of intracellular production of IFN-γ the media was supplemented with 5% fetal bovine serum, 10mM HEPES, 60U/mL rmIL-2 (Peprotech) and 10μg/mL of Brefeldin A (BFA, Sigma). Mouse CD4^+^ T cells were isolated from the spleen of 8-week to 4-months old TCR transgenic DO11.10 mice using a mouse CD4^+^ T cell isolation kit (Miltenyi Biotec), according to manufacturer’s instructions. Purity was analyzed by flow cytometry and was above 90%. Cells were then stimulated with mouse T-activator CD3/CD28 Dynabeads® (Invitrogen), according to manufacturer’s instructions. After Dynabeads® removal, cells were cultured for a resting period of 5–8 days in RPMI 1640 supplemented as described above in addition to 50μM 2-mercaptoethanol, 100μM sodium pyruvate, 10mM HEPES and 60U/mL rmIL-2. For confocal microscopy experiments, by the end of the resting period, live cells were sorted based on FSC-A/SSC-A parameters. For production of viral particles used for A20 B cell transduction, HEK-293T cells were transiently transfected by the Calcium Phosphate Method with 20μg of pEQPAM3 and 20μg of pMSCV-M2-IRES-Zeo, pMSCV-M2Y-IRES-Zeo, pMSCV-eGFP-M2-IRES-Zeo or pMSCV-eGFP-M2Y-IRES-Zeo. After transduction, B cells were cultured for two weeks in RPMI supplemented with 400μg/mL of zeocin. In the case of eGFP-M2/M2Y constructs, GFP+ cells were sorted after transduction and prior to culture with zeocin. All transductions were performed in the presence of 8μg/mL of polybrene.

### Flow Cytometry

Live resting mouse CD4^+^ T cells were sorted on a BD FACSaria flow cytometer at the end of the resting period, based on FSC-A/SSC-A parameters. After sorting, cells were immediately used for conjugation experiments. A20 B cells transiently expressing eGPF-M2 or eGFP-M2Y were sorted on a BD FACSaria flow cytometer for eGFP expressing cells, prior to their use. Splenocyte single cell suspensions were prepared from spleens recovered at 14dpi. Red blood cells were lysed in hypotonic NH_4_Cl. Splenocyte suspensions or cell lines were incubated with Fc block prior to staining, washed with 1x PBS, and stained for 20min with primary antibodies. Cells were washed and incubated for 20min with streptavidin, when appropriate. All samples were resuspended in FACS buffer (1xPBS, 2% FBS) and analyzed on LSR Fortessa (BD Biosciences) using DIVA software (BD Biosciences) for acquisition. For conjugation experiments, cells were analyzed in supplemented culture media, instead of FACS buffer. For IFN-γ intracellular measurements a FACSCalibur was used along with CellQuest software (Becton-Dickinson Immunocytometry Systems, San José, CA). For this experiment cells were fixed with 4% paraformaldehyde (PFA) and permeabilized with 0.2% triton X-100 prior to incubation with detecting antibody. All steps until incubation with PFA were performed in FACS buffer supplemented with BFA. T cells incubated for 5h with 100 ng/ml phorbol-12-myristate-13-acetate (PMA, Sigma) and 2 μg/ml ionomycin (Sigma) were used as positive control. Data was analyzed using Flowjo (Tree Star).

### Conjugation and Immunofluorescence

A20 B cells, stably expressing M2, M2Y, eGFP, eGPF-M2 or eGFP-M2Y, were pulsed overnight, or at least 2h, with the indicated concentrations of OVAp (OVA peptide 323–339; GenWay) in RPMI. Afterwards, A20 B cells and/or mouse CD4^+^ T cells were loaded with one of the following live dyes, as indicated for each experiment: CellTracker^TM^ Blue CMAC (7-amino-4-chloromethylcoumarin), CellTracker^TM^ Green CMFDA (5-chloromethylfluorescein diacetate), CellTrace^TM^ Far-Red DDAO-SE, CellTracker^TM^ Orange CMTMR ((5-(and-6)-(((4-chloromethyl)benzoyl)amino) tetramethylrhodamine) from Molecular Probes, Invitrogen. B and T cells were added in the indicated ratios, centrifuged for 1min to promote conjugate formation, and incubated for a period of time, as is specified. For confocal microscopy experiments, after conjugation, cells were gently resuspended, added to poly-L-lysine-coated coverslips (BD Biosciences) and incubated for the indicated time at 37°C. Cells were fixed with 4% paraformaldehyde and permeabilized with 0.2% Triton X-100. Samples were incubated with primary antibodies for 1h, followed by 1h of incubation with a species-specific secondary antibody, when necessary. DNA was detected with DAPI (Invitrogen). Coverslips were mounted in Mowiol (Fluka) or Fluoromount-G (Southern Biotec) and examined using a Carl Zeiss LSM 510 confocal microscope, with a Plan-Apochromat objective of 63x (1.4 oil). Analysis was performed using AimImageBrowser (Zeiss LSM data server). For flow cytometry experiments, after conjugation, cells were gently resuspended and analyzed on a LSR Fortessa flow cytometer (BD Biosciences).

### Image Quantification

Image quantification was performed in a blinded fashion, in a minimum of 45 randomly selected fields, from a total of three independent experiments. Distances of the MTOC to the center of the B-T_H_ contact zone and signal quantifications were measured using AimImageBrowser (Zeiss LSM data server, Zeiss). To evaluate the percentage of conjugates showing pTyr and/or IFN-γ polarization to the contact zone, only images with a minimum of three T cells were analyzed. Number of T cells conjugating was scored visually.

### Measurement of Calcium Fluxes

A20 B cells, loaded or not with OVAp, and mouse OVAp-specific CD4^+^ T cells were processed as described above. T cells were loaded with 5μM Indo-1 AM (Invitrogen) [46] and conjugated for 5min with A20 B cell lines. Before starting sample acquisition a baseline was set on the 405/525 emission ratio using Indo-1 loaded T cells. Ionomycin (Sigma) activated T cells were used as a positive control. Baseline was acquired for approximately 2min and samples were acquired for an additional 18min on a MoFlow cytometer.

### Enzyme Linked Immunosorbent Assay

A20 B cell lines were loaded, or not, with different concentrations of OVAp (aa 323–339) overnight. Cells were then conjugated with purified mouse CD4^+^ T cells as described above and incubated for 20h. After the 20h incubation period, supernatant was recovered and stored at -20°C. IFN-γ production was quantified in the supernatants by sandwich ELISA using the commercial kit DuoSet® ELISA Development System (R&D systems), according to manufacturer’s instructions. 96-well plates were analyzed on an Infinite® M200 (Tecan Group, Ltd). T cells cultured for two to four days in the presence of 3μg/mL of anti-CD3 antibody were used as positive control of T cell activation. B cells cultured for 48h in the presence of 2.5μg/mL of anti-CD40 antibody and 5μg/mL of the F(ab’)2/F(ab) portion of an anti-mouse IgG antibody were used as positive control for B cell activation.

### Statistical Analysis

Statistical significance was evaluated with unpaired one-tailed t-test, one-way ANOVA or non-parametric Mann-Whitney U test, as appropriate, using GraphPad Prism software. ns indicates p>0.05; * indicates p<0.05, ** indicates p<0.01, *** indicates p<0.001, **** indicates p<0.0001.

## Supporting Information

S1 Fig M2-expressing independent B cell line shows upregulation of CD80, CD86 and ICAM-1. (A) M2/M2Y expression in total cellular lysates of the independent A20 B cell lines. eGFP or eGFP-M2/M2Y fusion proteins were detected on Western Blot with an anti-eGFP antibody. An anti-actin antibody was used to demonstrate that similar amounts of cellular lysates were used. An A20 B cell line expressing non-tagged M2 (lane 4) was used as a negative control. (B) Fold increase of the mean fluorescence intensities (MFI) of several surface molecules, normalized to eGFP A20 B cells. A20 B cell lines stably expressing eGFP, eGFP-M2 or eGFP-M2Y were stained with fluorescently labelled antibodies and the surface expression of the indicated molecules was analyzed on a LSR Fortessa flow cytometer. Bars represent the mean of eight independent experiments. Error bars represent standard error of the mean. Statistical significance was assessed with a one-tailed Students t-test.
(TIF)

S2 Fig M2 expression in an independent B cell line promotes conjugation with T_H_ cells. eGFP independent B cell lines were pulsed overnight, or not, with different concentrations of OVAp and incubated with OVAp-specific CD4^+^ T cells at a 2:1 ratio. (A) Percentage of conjugates after 30min of incubation upon variation of the OVAp concentration. T cell populations were loaded with DDAO, to allow their discrimination. Results shown correspond to mean of three independent experiments. Statistical significance refers to comparison between M2 and M2Y conditions. (B) Percentage of conjugates per image after 30min of incubation, determined by confocal microscopy, upon variation of the OVAp concentration. Conjugate count was blind and based on B-T_H_ contact and pTyr polarization to the contact zone. 15 to 35 images were taken per sample, for an equivalent number of analyzed T cells within each OVAp concentration. Only images with a minimum of three T cells were considered for analysis. Results are from one experiment. (C) Fold increase of the number of conjugates formed with eGFP-M2- (open bars) or eGFP-M2Y- (filled bars) expressing B cells relative to M2Y condition. eGFP-M2-expressing B cells, eGFP-M2Y-expressing B cells and CD4^+^ T cells were mixed at a 1:1:1 ratio and incubated for 30min. Prior to conjugation M2Y-expressing B and T cell populations were labeled with the live dyes CMTMR and DDAO, respectively, to allow their discrimination. Conjugate formation was analyzed on a LSR Fortessa flow cytometer as the percentage of eGFP^+^DDAO^+^ (M2) or eGFP^+^CMTMR^+^DDAO^+^ (M2Y) events in the total DDAO^+^ population. (D) Representative FACS plots for each OVAp concentration. Percentage of T cells conjugating with M2- or M2Y-expressing B cells is indicated in the respective quadrant. In flow cytometry experiments, error bars represent standard error of the mean. Statistical significance between groups was evaluated by a one-tailed unpaired Student’s t test. In confocal microscopy experiments, statistical significance of the difference between groups was evaluated by a Mann-Whitney U test.
(TIF)

S3 Fig An independent M2-expressing B cell line requires specific peptide to promote T_H_ cell activation. (A) Average of the percentage of CD4^+^ T cells mobilizing calcium when conjugated with eGFP-M2-expressing (black bars), eGFP-M2Y-expressing (white bars) or eGFP-expressing (grey bars) B cells. eGFP independent B cell lines were pulsed overnight, or not, with different concentrations of OVAp and incubated with OVAp-specific CD4^+^ T cells for 5 min. Prior to conjugation T cells were loaded with Indo-I, a calcium indicator. Ionomycin was used as a positive control. Calcium fluxes were measured on a MoFlow cytometer for 21 minutes and were based on the 405/530 emission ratio over time. Graph shows results from one experiment. (B) Quantification of conjugates showing IFN-γ polarization to the contact zone per field. Prior to incubation B and T cells were labelled with CMFDA and CMAC live dyes, respectively. Cells were incubated for 2.5h, fixed and stained for IFN-γ and pTyr. Conjugates were evaluated by confocal microscopy based on B-T_H_ contact and IFN-γ polarization. Only images with a minimum of three T cells were considered for analysis. Statistical significance of the difference between groups was evaluated by a Mann-Whitney U test.
(TIF)
