# Correction: Murid Gammaherpesvirus Latency-Associated Protein M2 Promotes the Formation of Conjugates between Transformed B Lymphoma Cells and T Helper Cells

**DOI:** 10.1371/journal.pone.0145942

**Published:** 2015-12-22

**Authors:** Diana Fontinha, Filipa B. Lopes, Sofia Marques, Marta Alenquer, J. Pedro Simas

Dr. Marta Alenquer should be included in the author byline instead of the Acknowledgments. She should be listed as the fourth author, and her affiliation is: 1: Instituto de Medicina Molecular e Instituto de Microbiologia, Faculdade de Medicina, Universidade de Lisboa, Lisboa, Portugal. The contributions of this author are as follows: Performed the experiments.

The correct citation is: Fontinha D, Lopes FB, Marques S, Alenquer M, Simas JP (2015) Murid Gammaherpesvirus Latency-Associated Protein M2 Promotes the Formation of Conjugates between Transformed B Lymphoma Cells and T Helper Cells. PLoS ONE 10(11): e0142540. doi:10.1371/journal.pone.0142540
