# Supplementary figures and images for: Murid Gammaherpesvirus Latency-Associated Protein M2 Promotes the Formation of Conjugates between Transformed B Lymphoma Cells and T Helper Cells
Source: PLoS One. 2015 Nov 6;10(11):e0142540. doi: 10.1371/journal.pone.0142540 (PMC4636232; doi:10.1371/journal.pone.0142540)

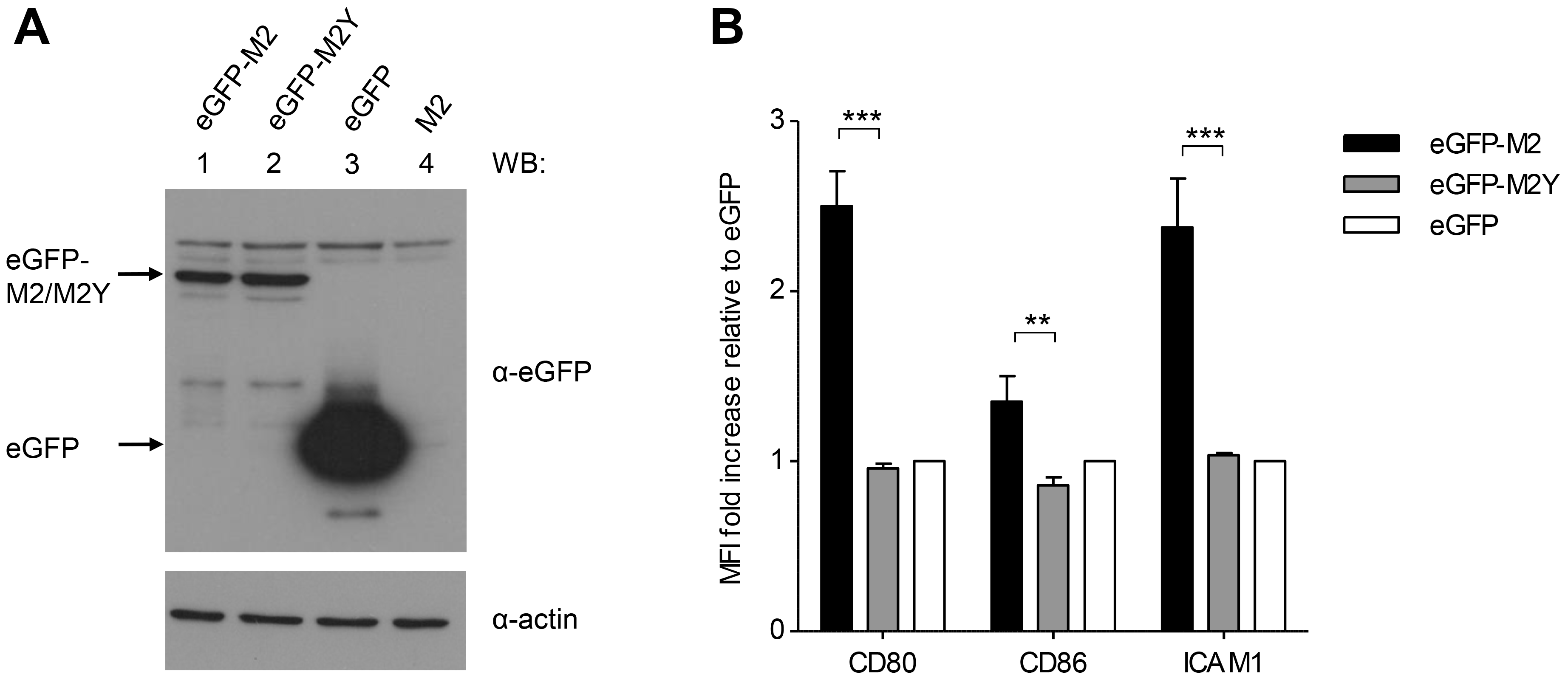

Supplement: S1 Fig — (A) M2/M2Y expression in total cellular lysates of the independent A20 B cell lines. eGFP or eGFP-M2/M2Y fusion proteins were detected on Western Blot with an anti-eGFP antibody. An anti-actin antibody was used to demonstrate that similar amounts of cellular lysates were used. An A20 B cell line expressing non-tagged M2 (lane 4) was used as a negative control. (B) Fold increase of the mean fluorescence intensities (MFI) of several surface molecules, normalized to eGFP A20 B cells. A20 B cell lines stably expressing eGFP, eGFP-M2 or eGFP-M2Y were stained with fluorescently labelled antibodies and the surface expression of the indicated molecules was analyzed on a LSR Fortessa flow cytometer. Bars represent the mean of eight independent experiments. Error bars represent standard error of the mean. Statistical significance was assessed with a one-tailed Students t-test. (TIF) [file pone.0142540.s001.tif]

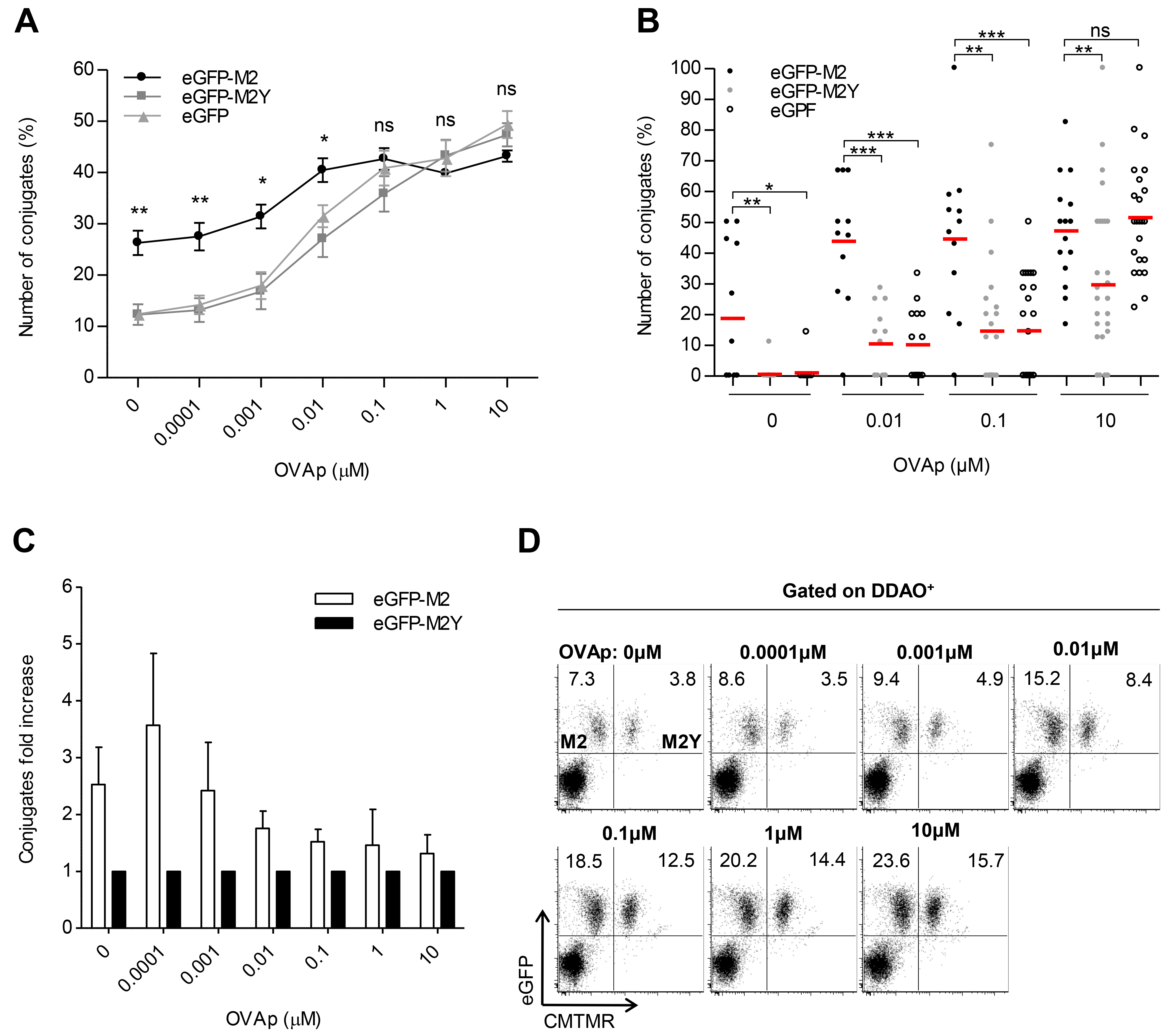

Supplement: S2 Fig — eGFP independent B cell lines were pulsed overnight, or not, with different concentrations of OVAp and incubated with OVAp-specific CD4+ T cells at a 2:1 ratio. (A) Percentage of conjugates after 30min of incubation upon variation of the OVAp concentration. T cell populations were loaded with DDAO, to allow their discrimination. Results shown correspond to mean of three independent experiments. Statistical significance refers to comparison between M2 and M2Y conditions. (B) Percentage of conjugates per image after 30min of incubation, determined by confocal microscopy, upon variation of the OVAp concentration. Conjugate count was blind and based on B-TH contact and pTyr polarization to the contact zone. 15 to 35 images were taken per sample, for an equivalent number of analyzed T cells within each OVAp concentration. Only images with a minimum of three T cells were considered for analysis. Results are from one experiment. (C) Fold increase of the number of conjugates formed with eGFP-M2- (open bars) or eGFP-M2Y- (filled bars) expressing B cells relative to M2Y condition. eGFP-M2-expressing B cells, eGFP-M2Y-expressing B cells and CD4+ T cells were mixed at a 1:1:1 ratio and incubated for 30min. Prior to conjugation M2Y-expressing B and T cell populations were labeled with the live dyes CMTMR and DDAO, respectively, to allow their discrimination. Conjugate formation was analyzed on a LSR Fortessa flow cytometer as the percentage of eGFP+DDAO+ (M2) or eGFP+CMTMR+DDAO+ (M2Y) events in the total DDAO+ population. (D) Representative FACS plots for each OVAp concentration. Percentage of T cells conjugating with M2- or M2Y-expressing B cells is indicated in the respective quadrant. In flow cytometry experiments, error bars represent standard error of the mean. Statistical significance between groups was evaluated by a one-tailed unpaired Student’s t test. In confocal microscopy experiments, statistical significance of the difference between groups was evaluated by a Mann-W [file pone.0142540.s002.tif]

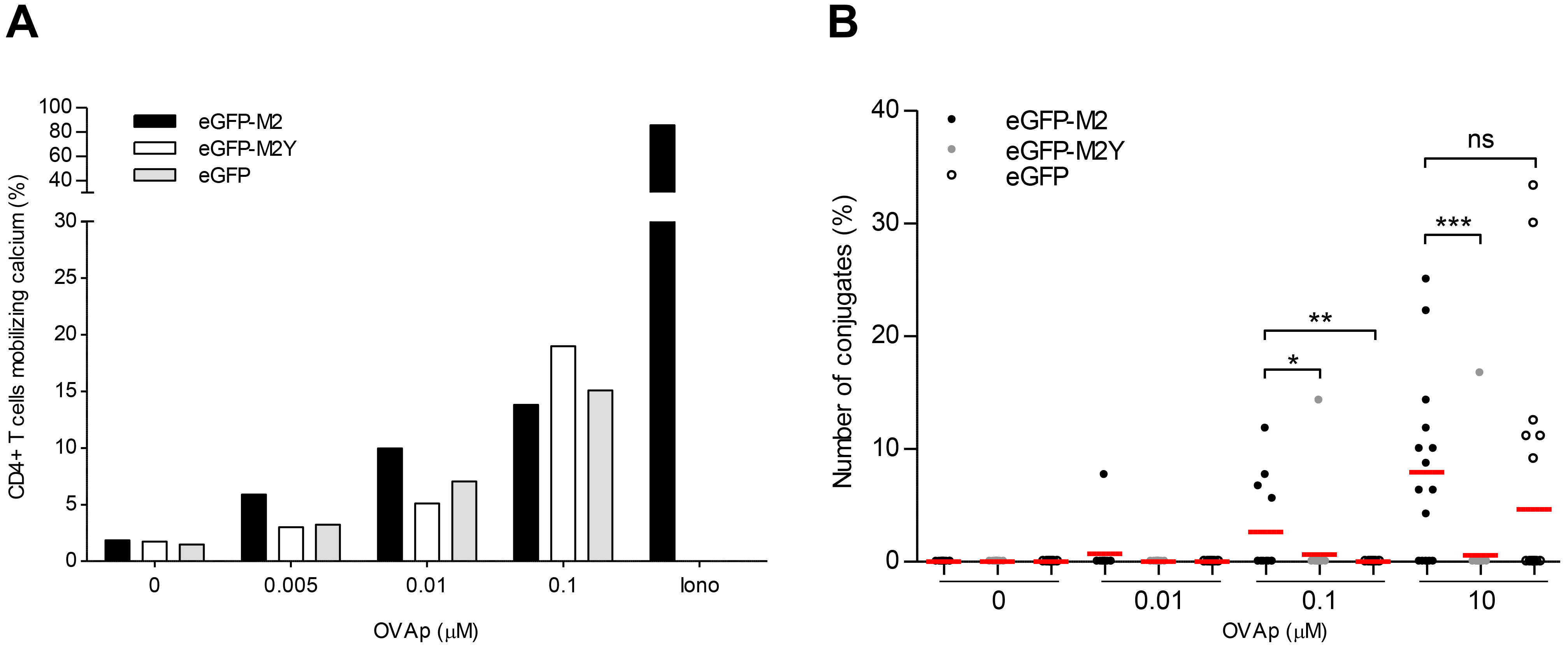

Supplement: S3 Fig — (A) Average of the percentage of CD4+ T cells mobilizing calcium when conjugated with eGFP-M2-expressing (black bars), eGFP-M2Y-expressing (white bars) or eGFP-expressing (grey bars) B cells. eGFP independent B cell lines were pulsed overnight, or not, with different concentrations of OVAp and incubated with OVAp-specific CD4+ T cells for 5 min. Prior to conjugation T cells were loaded with Indo-I, a calcium indicator. Ionomycin was used as a positive control. Calcium fluxes were measured on a MoFlow cytometer for 21 minutes and were based on the 405/530 emission ratio over time. Graph shows results from one experiment. (B) Quantification of conjugates showing IFN-γ polarization to the contact zone per field. Prior to incubation B and T cells were labelled with CMFDA and CMAC live dyes, respectively. Cells were incubated for 2.5h, fixed and stained for IFN-γ and pTyr. Conjugates were evaluated by confocal microscopy based on B-TH contact and IFN-γ polarization. Only images with a minimum of three T cells were considered for analysis. Statistical significance of the difference between groups was evaluated by a Mann-Whitney U test. (TIF) [file pone.0142540.s003.tif]
